# Transmission of SARS‐CoV‐2 through breast milk and breastfeeding: a living systematic review

**DOI:** 10.1111/nyas.14477

**Published:** 2020-08-28

**Authors:** Elizabeth Centeno‐Tablante, Melisa Medina‐Rivera, Julia L. Finkelstein, Pura Rayco‐Solon, Maria Nieves Garcia‐Casal, Lisa Rogers, Kate Ghezzi‐Kopel, Pratiwi Ridwan, Juan Pablo Peña‐Rosas, Saurabh Mehta

**Affiliations:** ^1^ Division of Nutritional Sciences Cornell University Ithaca New York; ^2^ Department of Maternal, Newborn, Child and Adolescent Health and Ageing World Health Organization Geneva Switzerland; ^3^ Department of Nutrition and Food Safety World Health Organization Geneva Switzerland; ^4^ Albert R. Mann Library Cornell University Ithaca New York

**Keywords:** COVID‐19, SARS‐CoV‐2, novel coronavirus, 2019 nCoV, severe acute respiratory syndrome, vertical transmission, perinatal transmission, mother‐to‐child transmission, breast milk, breastfeeding

## Abstract

The pandemic of coronavirus disease 2019 (COVID‐19) is caused by infection with a novel coronavirus strain, the severe acute respiratory syndrome coronavirus 2 (SARS‐CoV‐2). At present, there is limited information on potential transmission of the infection from mother to child, particularly through breast milk and breastfeeding. Here, we provide a living systematic review to capture information that might necessitate changes in the guidance on breast milk and breastfeeding given the uncertainty in this area. Our search retrieved 19,414 total records; 605 were considered for full‐text eligibility and no ongoing trials were identified. Our review includes 340 records, 37 with breast milk samples and 303 without. The 37 articles with analyzed breast milk samples reported on 77 mothers who were breastfeeding their children; among them, 19 of 77 children were confirmed COVID‐19 cases based on RT‐PCR assays, including 14 neonates and five older infants. Nine of the 68 analyzed breast milk samples from mothers with COVID‐19 were positive for SARS‐CoV‐2 RNA; of the exposed infants, four were positive and two were negative for COVID‐19. Currently, there is no evidence of SARS‐CoV‐2 transmission through breast milk. Studies are needed with longer follow‐up periods that collect data on infant feeding practices and on viral presence in breast milk.

## Graphical abstract

The objective of this review is to assess available evidence related to the possible transmission of SARS‐ CoV‐2 through breast milk and through breastfeeding (i.e., related bodily fluids, such as blood, sweat, and respiratory droplets) or droplet transmission due to close contact with the infant or young child via skin‐to‐skin exposure or airborne transmission. The review aims to support policymakers in making evidence‐informed global, regional, and national guidelines on infant feeding in the context of the ongoing pandemic.

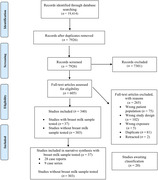

## Introduction

On March 11, 2020, the World Health Organization (WHO) declared the coronavirus disease (COVID‐19) outbreak a pandemic, with more than 600,000 cases globally.^1^ Infection with a novel coronavirus strain, SARS‐CoV‐2, causes COVID‐19. Clinical manifestation of SARS‐CoV‐2 infection varies; most infections are asymptomatic or present with mild symptoms, such as fatigue, fever, and cough.^2^ Severe cases may progress to viral pneumonia and require oxygen therapy, intensive care, and mechanical ventilation.^3^

There is limited but increasing evidence of SARS‐CoV‐2 infection during pregnancy. As initial reports on infections have been primarily from pregnant women confirmed or suspected with COVID‐19 who were infected in either the third or late second trimester of pregnancy, it is not yet entirely clear if pregnant women are at greater risk of becoming infected or if SARS‐CoV‐2 can be transmitted from mother to infant during pregnancy, delivery, or breastfeeding.^4^, ^5^ Similarly, information on COVID‐19 during infancy or in childhood remains sparse.

On May 27, 2020, WHO updated Interim Guidance on Clinical Management of COVID‐19, recommending exclusive breastfeeding for at least the first 6 months and breastfeeding alongside complementary foods until 2 years of age while using necessary precautions for infection prevention and control in infants born to mothers with suspected or confirmed COVID‐19 (WHO 2020a)^b^. This recommendation is based on the health benefits associated with breastfeeding for both the mother and the child and the relatively mild or asymptomatic illness experienced by infants reported so far. Infant feeding practices are an area of concern for families and health personnel, particularly during such outbreaks and high uncertainty of associated risks, such as its impact on food and nutrition security.

The objective of this review is to assess available evidence related to the possible transmission of SARS‐CoV‐2 through breast milk and breastfeeding (i.e., related bodily fluids, such as blood, sweat, and respiratory droplets) or droplet transmission due to close contact with the infant or young child via skin‐to‐skin exposure or airborne transmission. The review aims to support policymakers in making evidence‐informed global, regional, and national guidelines on infant feeding in the context of the ongoing pandemic.

## Methods

We designed and piloted a structured search strategy. The search was carried out on July 07, 2020, in the following electronic databases: MEDLINE (PubMed), the WHO COVID‐19 Global literature on coronavirus disease (https://search.bvsalud.org/global-literature-on-novel-co ronavirus‐2019‐ncov/), Cochrane Library, Web of Science Core Collection, and Embase (May 15, 2020). We also searched the COVID‐19 subset of the WHO International Clinical Trials Registry Platform (ICTRP) (July 07, 2020) to identify ongoing and unpublished studies. The WHO COVID‐19 global literature database is a comprehensive multilingual database on COVID‐19 updated daily (Monday through Friday) from searches of bibliographic databases, hand searching, and the addition of other expert‐referred scientific articles.

**Figure 1:**
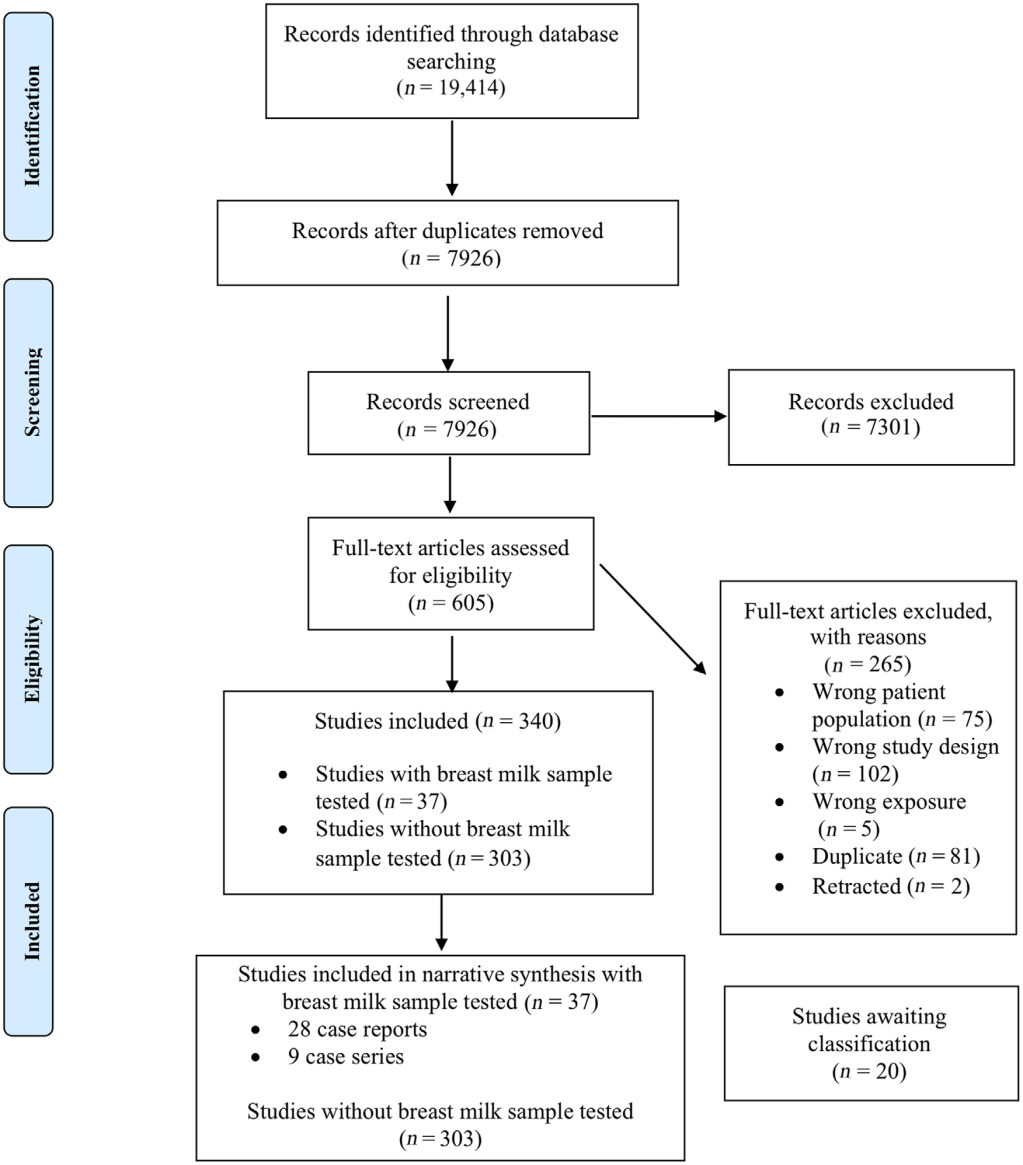
PRISMA chart (as of July 07, 2020).

Living systematic review guidelines employ a continual approach to searching the literature on rapidly emerging research topics, to ensure greater currency and validity, and to increase the benefits to end users.^6^ We used standard reporting methods described elsewhere.^7^ Updated searches are planned to be performed as needed to keep the results up to date as the COVID‐19 research base grows over time. Keywords used in the search strategy will be updated every week, incorporating new terminology for the virus as it comes into use. A detailed presentation of the number of studies retrieved, deduplicated, excluded during screening, and included in findings as of the third iteration of the search update is given in Figure 1 (PRISMA flow diagram). The protocol for this review is registered in PROSPERO, an international prospective register of systematic reviews: CRD42020178664.^8^

### Type of studies

We aimed to consider any study design, including case reports, case series, and a report of family clusters, which are part of the epidemiological data from the ongoing outbreak investigations. Other study designs, such as cohort studies, were considered for inclusion, but none were identified with the search strategy.

### Types of participants

Pregnant or lactating women with suspected, probable, or confirmed SARS‐CoV‐2 infection as well as their infants or young children (0–24 months of age) regardless of breastfeeding status, with suspected or confirmed SARS‐CoV‐2 infection, were eligible for inclusion. Case definitions are based on WHO Global surveillance interim guidance for COVID‐19 (WHO 2020c)^c^.

### Types of exposure

Apparently healthy infants or young children consuming breast milk directly from the breast or expressed breast milk from a woman with confirmed SARS‐CoV‐2 infection were considered exposed.

### Types of outcomes

The primary outcome was any infant with suspected, probable, or confirmed SARS‐CoV‐2 infection within 30 days of breastfeeding or receiving expressed breast milk from a woman with a suspected, probable, or confirmed SARS‐CoV‐2 infection. The secondary outcomes include the presence of SARS‐CoV‐2 RNA in breast milk by RT‐PCR, infant adverse effects, and neonatal mortality or morbidity.

### Search strategy

A comprehensive search strategy was designed to identify all available research pertaining to COVID‐19 and breastfeeding practices. An initial search was conducted (March 10 and 17, 2020) as part of rapid assessment of the evidence. On the second search (April 10, 2020), we included all known variations of terms to describe COVID‐19 at the time of searching (i.e., severe acute respiratory syndrome coronavirus 2, 2019nCoV, and SARS COV 2). Another iteration of the search was performed (July 07, 2020), and incorporated search terms for breastfeeding OR pregnancy OR mothers OR infants OR vertical transmission, to provide a more focused set of search results as the literature base on COVID‐19 rapidly grows in scope and volume. This latest version of the search strategy will be performed as needed going forward, and a 1‐week date filter will be applied to capture all new available literature that meets search criteria within the previous weeks’ time. The full search strategy in all its variations for the databases used is presented in the Supplementary Materials (online only). We plan to consider the following sources prior to each search of the literature, and updates to the search strategies will be made accordingly.

- The National Library of Medicine's Technical Bulletin: regular announcements regarding new Medical Subject Headings and synonyms for COVID‐19. (https://www.nlm.nih.gov/pubs/techbull/jf20/brief/jf20_mesh_novel_coronavirus_disease.html)
- The Medical Library Association's crowdsourced compilation of COVID‐19 search strategies (https://www.mlanet.org/p/cm/ld/fid = 1713)
- The Australian Library and Information Association's database of live COVID‐19 literature searches (https://www.alia.org.au/groups/HLA/covid-19-live-literature-searches).

### Study selection

The references captured by the search strategy were screened using the Covidence systematic review software (Veritas Health Innovation, Melbourne, Australia. Available at www.covidence.org). Two authors independently screened titles and abstracts according to the inclusion criteria; any discrepancies were resolved through discussion with a third author. The included full‐text articles were retrieved and managed using EndNote X9 (Thomson Reuters Corporation).

### Data extraction and management

Two authors independently extracted data from the included studies, using a piloted data extraction form. Since all the included studies were case reports or case series, it was not possible to calculate effect estimates, such as risk or odds ratio. For each study, information pertaining to exposure, diagnosis, symptoms, and infant feeding practices is described in the Results section.

### Quality of the evidence

The GRADE approach was followed for rating the quality of the evidence.^9^ Data on primary and secondary outcomes were considered: SARS‐CoV‐2 infection in infants and children breastfeeding from mothers with confirmed SARS‐CoV‐2 infection, and detection of SARS‐CoV‐2 in breast milk from mothers with confirmed SARS‐CoV‐2 infection. The GRADE approach included risk of bias, directness of evidence, inconsistency (heterogeneity), precision of effect estimates, and risk of publication bias across the included studies. We downgraded the certainty of the evidence according to study limitations (i.e., risk of bias, consistency, and directness of measurements of effects) and possible risk of publication bias, dose–response gradient, large effect, and other confounders. Serious or very serious limitations in any of these aspects led to a one‐ or two‐level downgrade in certainty. All the included reports are observational studies, which provide low‐quality evidence according to the GRADE approach. Therefore, the quality assessment is being presented in a narrative way instead of using summary of findings tables, which will be used in future updates, as more evidence becomes available.

## Results

Our search initially identified 19,414 records, of which, 7926 titles and abstracts were screened after removing duplicates. A total of 605 full‐text articles were assessed for eligibility, of which 265 were excluded. A total of 340 reports were included, of which 37 included the analysis of breast milk samples and 303 described lactating women or infants and children 0–24 months of age without collection or SARS‐CoV‐2 testing of breast milk samples. There were 20 records where the full text could not be located and are thus awaiting assessment.

Among the 303 reports without breast milk sample collection, there were 73 included reports that did not contribute data to inform the results of this review due to lack of breast milk or outcomes of interest: 36 reports presented pooled data of children of breastfeeding age and older; 27 other reports included pregnancy outcomes but not neonatal or breast milk outcomes; in three reports, neonates were not tested; six studies described ongoing pregnancies; and two reported a spontaneous abortion or pregnancy termination. References to these studies can be found in Table 2.

### Study designs

Twenty‐eight case reports,^10^, ^11^, ^12^, ^13^, ^14^, ^15^, ^16^, ^17^, ^18^, ^19^, ^20^, ^21^, ^22^, ^23^, ^24^, ^25^, ^26^, ^32^, ^33^, ^34^, ^36^, ^37^, ^38^, ^39^, ^40^, ^42^, ^43^, ^45^ and nine retrospective case series.^27^, ^28^, ^29^, ^30^, ^31^, ^35^, ^41^, ^44^, ^46^ These report articles were included for narrative analysis.

### Settings

The included studies were reports from Australia (*n* = 1),^32^ Belgium (*n* = 1),^33^ Canada (*n* = 1),^34^ China (*n* = 21),^12^, ^13^, ^18^, ^19^, ^20^, ^22^, ^23^, ^24^, ^25^, ^26^, ^27^, ^28^, ^29^, ^30^, ^31^, ^41^, ^42^, ^43^, ^44^, ^45^, ^46^ Germany (*n* = 2),^14^, ^35^ Italy (*n* = 6),^10^, ^11^, ^21^, ^36^, ^37^, ^38^ Jordan (*n* = 1),^39^ Singapore (*n* = 1),^17^ Turkey (*n* = 2),^16^, ^40^ and the Republic of Korea (*n* = 1).^15^ No additional information was available on the setting characteristics of the cases described.

Among the studies without breast milk samples, there were reports from Australia (*n* = 1), Belgium (*n* = 1), Brazil (*n* = 1), China (*n* = 122), France (*n* = 8), Germany (*n* = 5), Honduras (*n* = 1), India (*n* = 3), Iran (*n* = 7), Iraq (*n* = 2), Ireland (*n* = 1), Israel (*n* = 1), Italy (*n* = 26), Japan (*n* = 1), Jordan (*n* = 1), Lebanon (*n* = 1), Malaysia (*n* = 1), Mexico (*n* = 2), Morocco (*n* = 2), Netherlands (*n* = 3), Pakistan (*n* = 1), Peru (*n* = 1), Portugal (*n* = 1), Republic of Korea (*n* = 3), Spain (*n* = 17), Sweden (*n* = 3), Switzerland (*n* = 2), Thailand (*n* = 1), Turkey (*n* = 5), United Kingdom (*n* = 12), United States of America (*n* = 67), Venezuela (*n* = 1), and Vietnam (*n* = 1).

### Participants

The 37 reviewed studies reported on a total of 77 infants whose mothers were diagnosed with COVID‐19 and were able to provide a breast milk specimen for analysis. In these reports, there were 19 confirmed cases that included infants 2 years of age or younger. Among these cases, one newborn was diagnosed by the detection of anti‐SARS‐CoV–2–specific antibodies in serum.^12^ All the other cases were diagnosed by the detection of viral RNA by RT‐PCR from nasal or nasopharyngeal swabs or anal swabs (two cases).^35^ One study assessed breast milk samples for viral infection from five women with COVID‐19 but did not report on infant health outcomes or feeding practices for the corresponding five infants.^41^

Of the 19 positive children, 10 cases were reported to be breastfed and four mix‐fed from a woman with COVID‐19,^10^, ^14^, ^15^, ^17^, ^19^, ^21^, ^24^, ^25^, ^33^, ^34^, ^35^ two cases^12^, ^22^, ^32^, ^40^ were described to be fed with a breast milk substitute (infant formula), and two cases that did not provide a description of infant feeding practices.^11^, ^14^

Infection outcomes are summarized in Tables 1 and 2. Cases are presented as neonates (≤28 days old) and infants and young children (>28 days old to 24 months old) as these age groups potentially represent different exposure to breast milk and case management. Many neonates are tested regardless of symptoms due to maternal symptoms during pregnancy or the peripartum period, while older infants are less likely to be tested in the absence of severe symptoms.

**Table 1:** Children infection outcomes among articles with breast milk samples tested for SARS‐CoV‐2 RNA

Articles with breast milk samples available: 37				
Neonates ≤28 days old				
	Confirmed COVID‐19*a*	Negative COVID‐19*b*	Total	Studies
Breastfeeding*c*	8	15	23	Positive cases^14^, ^15^, ^21^, ^33^, ^34^, ^35^ Negative cases^16^, ^35^, ^37^, ^38^
Breast milk substitute (infant formula)^*d*^	2	16	18	Positive cases^12^, ^22^ Negative cases^13^, ^20^, ^26^, ^30^, ^36^, ^42^
Mix‐feeding	2	2	4	Positive cases^10^, ^40^ Negative cases^10^, ^39^
Not reported feeding practice	2	25	27	Positive cases^11^, ^14^ Negative cases^13^, ^18^, ^23^, ^27^, ^28^, ^29^, ^31^, ^43^, ^44^, ^45^
Infants >28 days old)				
Breastfeeding*c*	2	0	2	17, 25
Breast milk substitute (infant formula)*d*	0	0	0	
Mix‐feeding	3	0	3	19, 24, 32
Not reported feeding practice	0	0	0	
Total	19	58	77	

^*a*^ Confirmed cases are defined as individuals with a positive RT‐PCR test for SARS‐CoV‐2 RNA.

^*b*^ Negative COVID‐19 are individuals with a negative RT‐PCR test for SARS‐CoV‐2 RNA.

^*c*^ The frequency of exclusivity of breastfeeding before infection is not clear among these studies.

^*d*^ In one case,^12^ the newborn was isolated from the mother and, therefore, presumably fed with a breast milk substitute.

**Table 2:** Children infection outcomes among articles without breast milk samples tested

Articles without breast milk samples available: 303				
Neonates (0 to 28 days old)				
	Confirmed	Negative	Total	Studies
	COVID‐19*a*	COVID‐19*b*		
Breastfeeding*c*	16	137	153	Positive cases^60^, ^61^, ^62^, ^63^, ^64^, ^65^, ^66^, ^67^, ^68^, ^69^
				Negative cases^68^, ^70^, ^71^, ^72^, ^73^, ^74^, ^75^, ^76^, ^77^, ^78^, ^79^
Breast milk substitute (infant formula)*d*	15	67	82	Positive cases^62^, ^63^, ^80^, ^81^, ^82^, ^83^, ^84^, ^85^, ^86^, ^87^
				Negative cases^4^, ^68^, ^79^, ^81^, ^88^, ^89^, ^90^, ^91^, ^92^, ^93^, ^94^, ^95^, ^96^, ^97^, ^98^, ^99^, ^100^, ^101^, ^102^, ^103^
Mix‐feeding	3	7	10	Positive cases^104^, ^105^, ^106^
				Negative cases^107^
Not reported feeding practice	76	596	672	Positive cases^63^, ^67^, ^108^, ^109^, ^110^, ^111^, ^112^, ^113^, ^114^, ^115^, ^116^, ^117^, ^118^, ^119^, ^120^, ^121^, ^122^, ^123^, ^124^, ^125^, ^126^, ^127^, ^128^, ^129^, ^130^, ^131^, ^132^, ^133^, ^134^, ^135^, ^136^
				Negative cases^5^, ^10^, ^47^, ^75^, ^111^, ^114^, ^119^, ^120^, ^122^, ^137^, ^138^, ^139^, ^140^, ^141^, ^142^, ^143^, ^144^, ^145^, ^146^, ^147^, ^148^, ^149^, ^150^, ^151^, ^152^, ^153^, ^154^, ^155^, ^156^, ^157^, ^158^, ^159^, ^160^, ^161^, ^162^, ^163^, ^164^, ^165^, ^166^, ^167^, ^168^, ^169^, ^170^, ^171^, ^172^, ^173^, ^174^, ^175^, ^176^, ^177^, ^178^, ^179^, ^180^, ^181^, ^182^, ^183^, ^184^, ^185^, ^186^, ^187^, ^188^
Infants (>28 days old)				
Breastfeeding*c*	12	0	12	Positive cases^69^, ^189^, ^190^, ^191^, ^192^, ^193^, ^194^, ^195^, ^196^, ^197^, ^198^
Breast milk substitute (infant formula)*d*	6	0	6	Positive cases^83^, ^193^, ^199^, ^200^
Mix‐feeding	3	0	3	Positive cases^193^, ^201^
Not reported*e* feeding practice	125	2	127	Positive cases^48^, ^59^, ^85^, ^126^, ^202^, ^203^, ^204^, ^205^, ^206^, ^207^, ^208^, ^209^, ^210^, ^211^, ^212^, ^213^, ^214^, ^215^, ^216^, ^217^, ^218^, ^219^, ^220^, ^221^, ^222^, ^223^, ^224^, ^225^, ^226^, ^227^, ^228^, ^229^, ^230^, ^231^, ^232^, ^233^, ^234^, ^235^, ^236^, ^237^, ^238^, ^239^, ^240^, ^241^, ^242^, ^243^, ^244^, ^245^, ^246^, ^247^, ^248^, ^249^, ^250^, ^251^, ^252^, ^253^, ^254^
				Negative cases^155^, ^255^
Total	256	808	1065	
Articles not included in the analysis				3, 102, 115, 136, 178, 225, 256, 257, 258, 259, 260, 261, 262, 263, 264, 265, 266, 267, 268, 269, 270, 271, 272, 273, 274, 275, 276, 277, 278, 279, 280, 281, 282, 283, 284, 285, 286, 287, 288, 289, 290, 291, 292, 293, 294, 295, 296, 297, 298, 299, 300, 301, 302, 303, 304, 305, 306, 307, 308, 309, 310, 311, 312, 313, 314, 315, 316, 317, 318, 319, 320, 321, 322, 323, 324, 325, 326, 327, 328, 329, 330, 331, 332, 333, 334, 335, 336, 337, 338, 339, 340, 341, 342, 343, 344, 345, 346, 347, 348, 349, 350, 351, 352, 353, 354, 355

^*a*^ Confirmed cases are defined as individuals with a positive RT‐PCR tests for SARS‐CoV‐2 RNA.

^*b*^ Negative COVID‐19 are individuals with a negative RT‐PCR test for SARS‐CoV‐2 RNA.

^*c*^ The frequency of exclusivity of breastfeeding before infection is not clear among these studies.

^*d*^ There were 35 cases where authors clearly stated not breastfeeding, in 16 cases, authors advised not to breastfed, and 30 newborns were isolated at birth.

^*e*^ Among the cases with not reported feeding practices, there were 46 positive and 1 negative cases <1‐year‐old. There were 15 positive and 2 negative cases >1‐ to 2‐year‐old.

Among the included studies with laboratory‐confirmed breast milk samples from 77 mothers, the breastfed group composed of eight COVID‐19–positive neonates (≤28 days old),^10^, ^14^, ^15^, ^21^, ^33^, ^34^, ^35^ 15 neonates without COVID‐19,^16^ and two confirmed COVID‐19–positive infants 3 and 6 months old;^17^, ^25^ the mix‐fed group had two COVID‐19–positive neonate,^10^, ^40^ two negative neonates,^10^, ^39^ and three infants of 55 days and 8 and 14 months old;^19^, ^24^, ^32^ in the formula group, which includes infants separated from their mothers after birth, there were two positive infected neonates, 16 negative neonates,^13^, ^20^, ^26^, ^30^, ^36^, ^42^ and no infants.^12^, ^22^ Infant feeding practices were not reported for two positive neonates^11^, ^14^ and 25 neonates who were negative for COVID‐19.^13^, ^18^, ^23^, ^27^, ^28^, ^29^, ^31^, ^43^, ^44^, ^45^, ^46^

Three of the eight breastfed neonates with COVID‐19 developed signs and symptoms of respiratory illness,^14^, ^15^, ^21^ and one newborn required ventilation therapy and tube feeding.^14^ Two positive newborns^21^ were exclusively breastfed at the breast, the two mothers had positive nasopharyngeal swabs for COVID‐19 and presented mild symptoms. One of these neonates presented with cough, fever, diarrhea, and poor feeding and the other one was asymptomatic. In the case reported by Ref. 15, the 27‐day‐old neonate developed nasal stuffiness, fever, tachycardia, and jaundice during hospitalization. This child was breastfed from birth and her mother presented mild symptoms 1 day earlier; the grandparents who live in the same house showed symptoms a couple of days earlier and they were all positive for COVID‐19.

One case report^22^ described a neonate diagnosed with SARS‐CoV‐2 by RT‐PCR from a nasopharyngeal sample collected 36 h after birth; this neonate was never breastfed as a preventive measure to avoid transmission. The mother used a N95 mask (i.e., a particulate‐filtering facepiece respirator that filters at least 95% of airborne particles) during delivery and the neonate was separated 10 min after birth. The neonate did not have fever, difficulty breathing, or coughing; however, at 5 days of age, a chest computed tomography showed a high‐density nodular shadow under the pleura and lower lobe of the right lung. Six days later, there were small patchy shadows in the right lung, and after 5 more days, there were smaller pieces of patchy shadows. Both the mother and neonate recovered and were discharged without any other complications.^22^

Repeated elevated IgM and IgG levels were found in a neonate born to a mother diagnosed 23 days before birth with COVID‐19, the infant was delivered by cesarean section, the mother wore a N95 mask and did not hold the infant who was immediately quarantined.^12^ The infant was asymptomatic and there were no complications at birth. At birth, the infant's IgM level was 45.83 AU/mL and IgG 279.72 AU/mL and 14 days later, IgM levels were still elevated at 69.94 AU/mL and IgG 11.75 AU/mL suggesting *in utero* infection. Samples from the placenta or amniotic fluid were not analyzed and maternal vaginal secretions were negative by RT‐PCR test, as were five RT‐PCR tests of neonatal nasopharyngeal swabs taken between 2 and 13 days of life. The other 27 infants described in the included studies were negative for SARS‐CoV‐2 infection. The studies are briefly described in Table S1 (online only).

Of the eight breastfed infants, one 6‐month‐old infant did not develop any symptoms. Infants 3‐ and 14‐months of age developed mild symptoms and recovered completely.^19^, ^25^ In both cases, the infant's parents were positive for SARS‐CoV‐2. A 55‐day‐old infant^24^ presented with rhinorrhea and dry cough; during hospitalization, the chest computed tomography scan showed progressive pneumonia and laboratory results indicated alterations in hepatic function and abnormal myocardial zymogram. This infant was receiving mixed feeding before illness, and both parents and other family members were diagnosed with COVID‐19 2 days after infant symptoms onset. One neonate^33^ with confirmed COVID‐19 was born prematurely by cesarean section and transferred to a closed incubator. The mother presented symptoms on the day of birth and the infant's test was positive at 14 days of life. The infant was fed expressed breast milk and the infant did not present respiratory or gastrointestinal symptoms and continued in stable condition.

A total of 82 women provided breast milk samples for analysis. Nine out of 68 breast milk samples, collected from different mothers and assessed by an RT‐PCR assay, had detectable levels of SARS‐CoV‐2 RNA. Fourteen breast milk samples, from different women, were only assessed for the presence of specific antibodies, with one sample having specific IgG antibodies for SARS‐CoV‐2. The remaining 59 breast milk samples were negative. Of the six infants exposed to positive breast milk samples, two neonates were negative for viral RNA,^10^, ^31^ three neonates^14^, ^34^, ^40^ and one infant^32^ were positive for SARS‐CoV‐2 infection by RT‐PCR analysis. There were no infant outcomes reported for one mother with a positive breast milk sample.^41^

In one case report,^10^ a series of breast milk samples from the same women tested positive the day of delivery and on days 2 and 4 postpartum, and was negative on days 14–17. In the same case, viral RNA was also detected in placenta tissues and umbilical cord. The neonate nasopharyngeal swabs were negative at days 1, 3, 18, and 24 of life and the infant was clinically well.

Groß and collaborators^14^ described a neonate diagnosed with COVID‐19 who was exposed to breast milk with detectable SARS‐CoV‐2 by RT‐PCR, the breast milk samples tested positive during a 4‐day period, while the mother was symptomatic. Maternal symptoms initiated during the postpartum period and viral RNA was detected at day 8 postpartum, the neonate tested positive at day 11 and was also positive for syncytial virus infection, he presented with severe respiratory problems.^14^ The mother used a mask during the symptomatic period and followed hygiene recommendations while feeding and handling the neonate. There is no information about the infection status of other close relatives, the suspected close contact was a different mother–child pair with COVID‐19 sharing the same hospital room during the pre and postdelivery period.

In another study,^31^ SARS‐CoV‐2 RNA was detected in one of three breast milk samples from an asymptomatic mother. The mother was diagnosed with COVID‐19 the same day of delivery, the newborn was negative for viral infection by throat and anal swabs that were assessed on the first and third days after delivery. The infant feeding practices were not described.

In another case report,^32^ two out of six consecutively collected breast milk samples were positive for SARS‐CoV‐2 RNA, the positive samples were collected at 5 and 15 days after maternal symptoms. Maternal urine and saliva samples were negative for viral RNA. The 8‐month‐old infant had confirmed COVID‐19 with mild coryza symptoms and cough; breastfeeding was interrupted during the first 5 days of maternal symptoms and continued after the infant's diagnosis.

The time between maternal or infant symptoms and when breast milk samples were tested for SARS‐CoV‐2 RNA varied among the different studies. Most of the cases were tested during the acute phase, 1–5 days after maternal symptoms^14^, ^18^, ^22^, ^26^, ^29^, ^31^, ^34^, ^36^, ^38^, ^39^, ^40^, ^41^, ^45^ or between 5 and 8 days after onset of maternal symptoms.^10^, ^13^, ^16^, ^20^, ^32^, ^33^, ^37^ Other samples were collected during the convalescent phase at 14–19 days after maternal^17^, ^24^, ^29^, ^42^ or infant onset of symptoms.^25^ The time between maternal symptoms and breast milk sample collection was not reported in four neonates with COVID‐19^21^, ^35^ and other 25 neonates without infection.^28^, ^35^, ^44^

In another case,^12^ maternal symptoms occurred during pregnancy, at 34 gestational weeks, and the breast milk sample was collected 23 days later after delivery. Similarly, another pregnant woman was diagnosed with COVID‐19 during the 33rd gestational week, and a breast milk sample was tested on the day of delivery at 38 gestational weeks.^23^

The overall certainty of the evidence was very low for all included studies. The studies were all observational and included case reports and case series. All studies had a high risk of bias due to lack of a control group, short follow‐up time, and lack of control for other possible confounders. All of the included studies also had a high risk of imprecision since case reports and case series document outcomes on few cases. Additionally, the description of feeding practices was incomplete. We consider that a high risk of publication bias is plausible given that COVID‐19–positive cases of infants and breast milk are most likely to be reported and published. Additionally, the sample could also be biased by only testing infants who seek care and present with severe symptoms.

### Studies without breast milk sample assessment

A total of 303 reports on infants and young children 0–24 months of age did not test breast milk samples for SARS‐CoV‐2. We grouped these reports by the infant feeding practices (Table 2).

We identified 153 breastfeeding neonates, which included 16 positive SARS‐CoV‐2 infections and 137 negative cases, based on the lack of viral RNA detection by RT‐PCR tests in throat or nasopharyngeal samples. There were 82 neonate cases who were reportedly fed with a breast milk substitute or presumably so since some of the newborns were isolated from their mothers or the authors recommended stopping breastfeeding practices during hospital stay. Among these neonates, there were 15 positive cases and 67 negatives by RT‐PCR tests from throat or nasopharyngeal swabs. There were 10 cases of reportedly mix‐feeding practices: three of these neonates were positive and seven were negative for SARS‐CoV‐2 infection according to the lack of viral RNA detection by RT‐PCR test on oral or nasopharyngeal samples. A total of 672 neonate cases did not report on infant feedings. These included 76 positive neonates and 596 negative neonates for SARS‐CoV‐2 infection; neonate diagnosis was based on viral RNA detection in nasal or nasopharyngeal samples, except 19 neonates^47^ who were diagnosed as negative COVID‐19 cases based on clinical diagnosis. The references to these studies are listed in Table 2.

There were also 12 breastfeeding infants (of 28 days to 2 years of age) who were positive for SARS‐CoV‐2 infection, as well as other six infants fed with a breast milk substitute and three cases who were mix‐fed. There were no negative cases among these feeding categories. Infant feeding practices were not reported in 127 cases of infants, which included 112 infants with a positive diagnosis for COVID‐19 and two were negative cases (Table 2). Infant cases of COVID‐19 were determined by viral RNA detection with RT‐PCR assays from nasal or nasopharyngeal swabs, except by one infant who was diagnosed by clinical presentation.^48^

## Discussion

In this review, we assess the evidence on transmission of SARS‐CoV‐2 from the mother to her child through breast milk and breastfeeding (i.e., related bodily fluids, such as blood, sweat, and respiratory droplets) or droplet transmission due to close contact with the infant or young child via skin‐to‐skin exposure or airborne transmission. We also summarize outcomes for infants and children with suspected or confirmed SARS‐CoV‐2 infection according to breastfeeding practices, as reported by the authors.

Among the 37 included studies with breast milk samples, nine out of 84 analyzed breast milk samples were reported to be positive for SARS‐CoV‐2 RNA via RT‐PCR analysis^10^, ^14^, ^31^ and one sample had specific IgG.^35^ Among the cases with viral RNA detected in breast milk samples, one healthy neonate^10^ had negative nasopharyngeal and anal test results for COVID‐19, while maternal breast milk samples had detectable viral RNA. However, this neonate was fed with a breast milk substitute, while maternal breast milk was positive and thus, it is not possible to ascertain the risk of infection by exposure to breast milk. In another case, the infant tested negative to the virus and exposure through breast milk could not be confirmed because infant feeding practices were not reported.^31^ In a different case, a neonate was found to be positive for COVID‐19 based on viral RNA detected by RT‐PCR, while exposed to maternal breast milk that tested positive for SARS‐CoV‐2 RNA.^14^ The mother breastfed the neonate while using surgical masks and following hygiene recommendations. In this particular case, before showing symptoms, both mother and newborn shared a hospital room with another mother–infant pair diagnosed with COVID‐19. Thus, it is not clear whether the newborn became exposed due to close contact with the other confirmed COVID‐19 patients, contact with the confirmed COVID‐19–positive mother, or through the exposure to her SARS‐CoV‐2 RNA–positive breast milk. Moreover, this newborn was also coinfected with respiratory syncytial virus and it is possible that the coinfection could have increased the neonate's vulnerability to COVID‐19 or worsen the symptoms of the infection. In the case of an 8‐month‐old infant with COVID‐19,^32^ it is not possible to determine if transmission occurred through breast milk intake or breastfeeding, since it was interrupted during the maternal symptomatic phase, and the infant had symptoms 1 day after the mother's symptoms; additionally, mother and infant were staying in an area with ongoing COVID‐19 community transmission for 2 months.

Even though viral RNA has been detected in breast milk samples, among the included studies there were no attempts to culture the SARS‐CoV‐2 from breast milk isolates, adding to the uncertainty about potential infectious capacity of breast milk. One preprint study^49^ found no culturable virus from one breast milk sample with viral RNA and from control breast milk samples spiked with SARS‐CoV‐2.

From the two reports that described breastfeeding practices, it is not possible to conclude if SARS‐CoV‐2 infection was due to mother‐to‐child transmission, which can include but is not limited to specific breastfeeding practices or to feeding young children with breast milk from a COVID‐19–positive woman. For the latter, the evidence gathered also includes another 79 breast milk samples, belonging to different women with confirmed COVID‐19, which had no traces of SARS‐CoV‐2 RNA, and this may suggest a low risk of transmission by ingesting breast milk. Interestingly, there were several confirmed COVID‐19 infants by RT‐PCR tests who received SARS‐CoV‐2 negative breast milk; these included two newborns exclusively breastfed,^21^ one 27‐day‐old neonate who was breastfed,^15^ and infants aged 3 and 6 months old.^17^, ^25^ Therefore, it seems plausible that these infants might have been exposed to SARS‐CoV‐2 through close contacts with infected family members, especially considering that in all these cases, both parents, relatives, and individuals in their communities were diagnosed with COVID‐19. Notably, infant feeding practices were scarcely reported in most of the included studies.

From the evidence reported in articles without breast milk samples tested, it is not possible to ascertain if there is an increased risk of viral infection among breastfeeding children via breast milk. Most breastfeeding neonates did not have evidence of COVID‐19 (28 out of 33) based on negative RT‐PCR tests. Similarly, among infants who were fed with a breast milk substitute, 63 out of 77 did not have COVID‐19 based on negative RT‐PCR tests. However, infant feeding practices were not accurately reported. From all these studies, the frequency or exclusivity of breastfeeding is unclear, especially in relation to onset of maternal symptoms or viral load.

Most neonates born to mothers diagnosed with COVID‐19 by RT‐PCR tests during their pregnancy were negative for viral infection. One neonate was reported to be negative for COVID‐19 by RT‐PCR tests in throat swabs but had elevated IgM and IgG levels at birth.^12^ These findings may suggest that vertical transmission does not appear to occur during the peripartum period. It is possible that passive immunity from the mother to the infant could be protective against infection. This was also suggested in the context of severe acute respiratory syndrome coronavirus (SARS‐CoV) infection.^50^ Given that all maternal infections reported in the included studies occurred shortly before delivery, the consequences of infection during earlier stages of pregnancy are not clear. Additionally, the majority of included studies did not assess the presence of SARS‐CoV‐2–specific antibodies in neonates, breast milk, placenta, or other tissues and hence, the significance of finding anti‐SARS‐CoV‐2 IgM immediately after birth in serum samples from the neonates, as reported by Ref. 12, remains to be defined. It is also important to understand the potential of passive immunity from mother to child during pregnancy and by breast milk intake. Specific IgA antibodies have been found in 12 of 15 breast milk samples, for example, from different women who had COVID‐19,^51^ and further research is needed to ascertain the protective capacity and the duration of these antibodies.

It is also important to understand if the risk of transmission through breast milk changes in different stages of maternal disease progression or in asymptomatic cases. The three breast milk samples with detectable viral RNA were collected during the maternal symptomatic phase.^10^, ^14^, ^31^ But the 43 breast milk samples without detectable viral RNA were collected during either the symptomatic or convalescent phase. This might suggest that SARS‐CoV‐2 does not always cross the alveolus or milk‐secreting unit during the acute phase, when maternal viremia is expected to be higher, or during the convalescent phase. Even after 4–5 weeks of maternal infection, viral RNA was not detected in breast milk, possibly indicating that the alveolus does not act as a reservoir for SARS‐CoV‐2.

Breast milk is not homogenous and its composition changes through the lactation period. In the future studies, it will be important to determine if the risk of transmission changes with the stage of lactation. In this review, most of the breast milk samples (43 out of 46) were negative for SARS‐CoV‐2 RNA presence, and the majority were tested during the first 48 h postpartum. This early period usually has a higher concentration of immune factors in milk compared with the composition of mature milk after the first month postpartum. There were few samples at different stages indicating the need to further characterize the risk of SARS‐CoV‐2 transmission in the context of breast milk composition, especially considering the immunomodulating components of breast milk, including antibodies, growth factors, and other proteins, with critical roles in sustaining healthy neonatal intestinal epithelium and with antimicrobial properties.^52^ Lactoferrin, an iron‐binding protein present in breast milk, for example, has been found to inhibit SARS‐coronavirus infection in cell culture conditions.^53^

## What is known from SARS‐CoV

When we consider the past experience with SARS‐CoV, the viral agent that caused a major outbreak in 2003, it is important to note that it is genetically similar to SARS‐CoV‐2.^54^ We identified two case reports where SARS‐CoV was tested in breast milk.^55^ In one case,^56^ the breast milk sample collected at birth was positive for SARS‐CoV. The infant was exclusively breastfed and did not show any symptoms of the disease at 5 days of age. The mother was diagnosed with SARS‐CoV infection at 20 gestational weeks, and she required intensive care. In a second case report,^55^ the mother presented with serious symptoms of SARS‐CoV infection at 20 gestational weeks, and the infant was born healthy at 38 gestational weeks by cesarean section. Viral RNA was not detected in maternal samples of serum, whole blood, nasopharyngeal, and rectal swabs, or postdelivery, in the placenta, cord blood, amniotic fluid, and breast milk. However, antibodies to SARS‐CoV were detected in maternal serum, cord blood, and breast milk, suggesting that the newborn might have acquired passive immunity from the mother. Curiously, in both cases, the mothers developed gestational diabetes.^55^, ^56^

Another case series^57^ reported five infants born to mothers with SARS‐CoV infection and pneumonia. All five infants tested negative for the presence of SARS‐CoV RNA in samples of serum, throat swab, and urine. They also had negative cultures, but serum samples indicated the presence of SARS‐CoV antibodies. The antibody titres did not increase in paired samples between the acute (1–9 days) and convalescent phases (21–23 days), suggesting passive immune transfer during pregnancy.^57^ It is possible that the vertical transmission of SARS‐CoV may be prevented by the presence of antibodies in cord blood and breast milk, as previously described.^55^, ^57^ In summary, we reviewed the evidence for the transmission of the related SARS coronavirus (SARS‐CoV) and found that most studies reporting on infection status of mothers and their children did not describe breastfeeding behaviors or test breast milk samples. This evidence from the SARS‐CoV outbreak in 2003 can be useful in the interpretation of the results from this review on COVID‐19.

## Limitations

Findings from this review are limited by the scarcity of information of infant feeding practices and breast milk samples tested, there was very limited information on skin‐to‐skin contact, rooming‐in, and exclusive breastfeeding. In most cases, there was not clear information on the breast milk sample collection and analysis procedure. We also identified several studies from diverse countries that reported infant infection outcomes, but the reporting of feeding practices was heterogeneous or not available. Additionally, there could be a high risk of publication and sample bias, given that the most severe cases are the ones that seek care, are tested, and most commonly only those with a positive diagnosis are most likely to be reported. Moreover, there could be duplication of reported cases as case reports become part of a larger body of evidence. We plan to update these review findings as the evidence continues to grow with upcoming results from cohort studies and other case reports.

## Implications for future research

More evidence about the intake of breast milk directly from the breast or expressed, as well as infants being breastfed exclusively, or formula fed will further the understanding of SARS‐CoV‐2 transmission. Despite the rapidly increasing literature around SARS‐COV‐2 transmission routes, the evidence of possible transmission through breast milk is still limited. Most of the reported cases are symptomatic infants or symptomatic pregnant women and their newborns, the information on older infants with or without symptoms is scarce. In most cases, only one breast milk sample was tested and with no information on maternal viremia. Breast milk samples were only tested by RT‐PCR assays, and it is possible that viral RNA detection in breast milk was affected by the component of breast milk tested, as it has been shown to affect the assay sensitivity in other contexts.^58^ It is important to note that viral RNA detection in breast milk does not necessarily indicate viral infectivity, and other assays will be necessary to determine if there are viral particles in the breast milk that can be infectious once ingested by children.

This systematic review included findings from 37 studies; 19 infants out of 77 were diagnosed with COVID‐19 by viral RNA detection and one by serology; 59 breast milk samples that were collected from COVID‐19–positive mothers tested negative for SARS‐CoV‐2 via RT‐PCR. As new evidence emerges from the current SARS‐CoV‐2 outbreak, additional research is warranted to explore the possible dynamic of breastfeeding transmission or protection against SARS‐CoV‐2. Specifically, studies focused on defining the timing of maternal and infant exposure, breast milk viral load, duration of infection, and the presence of protective antibodies in breast milk might aid in determining the risks of SARS‐CoV‐2 transmission when a breastfeeding woman is infected. We suggest that investigators working with breastfeeding mothers and their children collect the following data, where feasible, to better inform this review and the consequent guidelines: (1) confirmation and clinical characteristics of maternal and infant infection; (2) analyze and report on the presence of virus and viral infectious particles in breast milk, potentially with serial samples; and (3) record data on infant feeding practices and any contact precautions observed, such as wearing a mask and isolation.

The review authors are uncertain whether SARS‐CoV‐2 transmission via breast milk is possible as the certainty of the evidence has been assessed as very low. Nonetheless, the possible transmission through other breastfeeding‐related bodily fluids, such as blood, sweat, respiratory droplets, or droplet transmission due to close contact with the infant or young child via skin‐to‐skin exposure or airborne transmission cannot be disregarded.

*Note adding in proof*. Since this work was completed and accepted, Chambers *et al*. report that although SARS‐CoV‐2 RNA was detectable in 1 breast milk sample from 18 infected woman, this did not represent replication‐competent virus; the authors concluded that breast milk may not be a source of infection for infants. *JAMA*. Published online August 19, 2020.https://doi.org/10.1001/jama.2020.15580

## Disclaimer

The authors alone are responsible for the views expressed in this review and they do not necessarily represent the views, decisions, or policies of the institutions with which they are affiliated.

## Author contributions

S.M., P.R.S., J.L.F., and J.P.P.R. conceptualized the study. E.C.T., M.M.R., J.L.F., P.R.S., M.N.G.C., L.R., J.P.P.R., and S.M. designed the protocol. K.G.K. and E.C.T. designed, updated, and translated the search strategy. E.C.T., M.M.R., and P.R. screened and extracted data from articles. E.C.T., M.M.R., J.L.F., P.R.S., M.N.G.C., L.R., P.R., J.P.P.R., and S.M. interpreted the findings. E.C.T. and M.M.R. wrote the first draft, which all authors revised for critical content. E.C.T. and M.M.R. contributed equally to this study. All authors approved the final manuscript. S.M. is the guarantor. The corresponding authors attest that all listed authors meet authorship criteria and that no others meeting the criteria have been omitted.

## Funding

This work was supported by the Division of Nutritional Sciences, Cornell University, and the World Health Organization, Geneva, Switzerland.

## Supporting information

Supplementary Material

Supplementary Material

## Footnotes

b WHO. Clinical management of severe acute respiratory infection (SARI) when COVID‐19 disease is suspected: interim guidance. 3 March 2020. World Health Organization; 2020.

c WHO. Global surveillance for COVID‐19 caused by human infection with COVID‐19 virus. Interim guidance. 20 March 2020.: World Health Organization; 2020.
