# Supplementary material for: Transmission of SARS‐CoV‐2 through breast milk and breastfeeding: a living systematic review
Source: Ann N Y Acad Sci. 2020 Aug 28;1484(1):32–54. doi: 10.1111/nyas.14477 (PMC7970667; doi:10.1111/nyas.14477)
Supplement: Supplementary file 2 — Supplementary Material [file NYAS-1484-32-s002.docx]

**Transmission of Novel Coronavirus (COVID-19) through breast milk and breastfeeding
*A living systematic review***

Full search strategies for searches performed up to May 15, 2020

**PubMed (MEDLINE)**

1. (Breast Feeding[Mesh] OR Milk, Human[Mesh] OR Lactation[Mesh] OR colostrum[mesh] OR Breastfe*[tiab] OR breast fe*[tiab] OR breastmilk[tiab] OR breast milk[tiab] OR human milk[tiab] OR maternal milk[tiab] OR prelacteal feed*[tiab] OR lactati*[tiab] OR colostrum[tiab] OR mixed feeding[tiab] OR **mother’s milk[tiab] OR expressed milk[tiab] OR milk bank*[tiab]**)
2. (Pregnant Women [Mesh] OR Pregnancy [Mesh] OR Prenatal Care [Mesh] OR Mothers[Mesh] OR infant[MeSH] OR pregnan*[tiab] OR gestat*[tiab] OR perinatal[tiab] OR antenatal[tiab] OR parturi*[tiab] OR mother*[tiab] OR infant*[tiab] OR maternal[tiab] OR baby[tiab] OR babies[tiab] OR newborn*[tiab] OR neonat*[tiab])
3. (Infectious Disease Transmission, Vertical[MeSH] OR vertical transmission[tiab] OR postnatal transmission[tiab] OR post natal transmission[tiab] OR maternal-infant transmission[tiab] OR adult-to-child[tiab] OR maternal-to-child[tiab] OR mother-to-child[tiab] OR MTCT[tiab] OR PMTCT[tiab])
4. #1 OR #2 OR #3
5. (COVID-19[Supplementary Concept] OR COVID-19 diagnostic testing[Supplementary Concept] OR severe acute respiratory syndrome coronavirus 2[Supplementary Concept] OR severe acute respiratory syndrome coronavirus 2[tiab] OR COVID-19[tiab] OR COVID19[tiab] OR COVID2019[tiab] OR 2019 ncov[tiab] OR 2019-nCoV[tiab] OR 2019nCoV[tiab] OR SARS COV 2[tiab] OR SARS-CoV-2[tiab] OR SARS2[tiab] OR hcov19[tiab] OR hcov2019[tiab] OR hcov-19[tiab] OR hcov-2019[tiab**]** OR wuhan coronavirus*[tiab] OR coronavirus disease 2019[tiab] OR coronavirus disease-19[tiab] OR SARS2[tiab] OR novel coronavirus*[tiab] OR novel corona virus*[tiab] OR new coronavirus*[tiab] OR new corona virus*[tiab] OR **COVID 2019[tiab]**)
6. #4 AND #5 AND Filters activated: Publication date from 2020/04/17 to 2020/12/3

**Web of Science Core Collection (Clarivate Analytics)**

1. TS=("Breastfe*" OR "breast fe*" OR "breastmilk" OR "breast milk" OR "human milk" OR "maternal milk" OR "prelacteal feed*" OR "lactati*" OR "colostrum" OR "mixed feeding" OR "**mother’s milk**" **OR** "**expressed milk**" **OR** "**milk bank***")
2. TS=("pregnan*" OR "gestat*" OR "perinatal" OR "antenatal" OR "parturi*" OR "mother*" OR "infant*" OR "maternal" OR "baby" OR "babies" OR "newborn*" OR "neonat*")
3. TS=("vertical transmission" OR "postnatal transmission" OR "post natal transmission" OR "maternal-infant transmission" OR "adult-to-child" OR "maternal-to-child" OR "mother-to-child" OR "MTCT" OR "PMTCT")
4. #1 OR #2 OR #3
5. TS=("severe acute respiratory syndrome coronavirus 2" OR "COVID-19" OR "COVID19" OR "COVID2019" OR "2019 ncov" OR "2019-nCoV" OR "2019nCoV" OR "SARS COV 2" OR "SARS-CoV-2" OR "SARS2" OR "hcov19" OR "hcov2019" OR "hcov-19" OR "hcov-2019" OR "wuhan coronavirus*" OR "coronavirus disease 2019" OR "coronavirus disease-19" OR "SARS2" OR "novel coronavirus*" OR "novel corona virus*" OR "new coronavirus*" OR "new corona virus*" OR "**COVID 2019**")
6. #4 AND #5 AND Filter: Publication date 1/2020-12/2020

**Cochrane Library (Cochrane)**

1. MeSH descriptor: [Breast Feeding] explode all trees
2. MeSH descriptor: [Milk, Human] explode all trees
3. MeSH descriptor: [Lactation] explode all trees
4. MeSH descriptor: [Colostrum] explode all trees
5. ("Breastfe*" OR "breast fe*" OR "breastmilk" OR "breast milk" OR "human milk" OR "maternal milk" OR "prelacteal feed*" OR "lactati*" OR "colostrum" OR "mixed feeding" OR "**mother’s milk**" **OR** "**expressed milk**" **OR** "**milk bank***"):ti,ab,kw
6. #1 OR #2 OR #3 OR #4 OR #5
7. MeSH descriptor: [Pregnant Women] explode all trees
8. MeSH descriptor: [Pregnancy] explode all trees
9. MeSH descriptor: [Prenatal Care] explode all trees
10. MeSH descriptor: [Mothers] explode all trees
11. MeSH descriptor: [Infants] explode all trees
12. ("pregnan*" OR "gestat*" OR "perinatal" OR "antenatal" OR "parturi*" OR "mother*" OR "infant*" OR "maternal" OR "baby" OR "babies" OR "newborn*" OR "neonat*"):ti,ab,kw
13. #7 OR #8 OR #9 OR #10 OR #11 OR #12
14. MeSH descriptor: [Infectious Disease Transmission, Vertical] explode all trees
15. ("vertical transmission" OR "postnatal transmission" OR "post natal transmission" OR "maternal-infant transmission" OR "adult-to-child" OR "maternal-to-child" OR "mother-to-child" OR "MTCT" OR "PMTCT"):ti,ab,kw
16. #14 OR #15
17. #6 OR #13 OR #16
18. ("severe acute respiratory syndrome coronavirus 2" OR "COVID-19" OR "COVID19" OR "COVID2019" OR "2019 ncov" OR "2019-nCoV" OR "2019nCoV" OR "SARS COV 2" OR "SARS-CoV-2" OR "SARS2" OR "hcov19" OR "hcov2019" OR "hcov-19" OR "hcov-2019" OR "wuhan coronavirus*" OR "coronavirus disease 2019" OR "coronavirus disease-19" OR "SARS2" OR "novel coronavirus*" OR "novel corona virus*" OR "new coronavirus*" OR "new corona virus*" **OR "COVID 2019"**):ti,ab,kw
19. #17 AND #18 AND Date range 2020 only

**EMBASE (Ovid)**

1. Exp breast feeding/ OR exp breast milk/ OR lactation/ OR exp colostrum/
2. ("Breastfe*" OR "breast fe*" OR "breastmilk" OR "breast milk" OR "human milk" OR "maternal milk" OR "**mother’s milk**" OR "prelacteal feed*" OR "lactati*" OR "colostrum" OR "mixed feeding" OR "mother’s milk" OR "expressed milk" OR "milk bank*").mp
3. #1 OR #2
4. Exp Pregnant woman/ OR exp pregnancy/ OR exp prenatal care/ OR exp mother/ OR exp infant
5. ("pregnan*" OR "gestat*" OR "perinatal" OR "antenatal" OR "parturi*" OR "mother*" OR "infant*" OR "maternal" OR "baby" OR "babies" OR "newborn*" OR "neonat*").mp
6. #4 OR #5
7. Exp disease transmission/
8. ("vertical transmission" OR "postnatal transmission" OR "post natal transmission" OR "maternal-infant transmission" OR "adult-to-child" OR "maternal-to-child" OR "mother-to-child" OR "MTCT" OR "PMTCT").mp
9. #7 OR #8
10. #3 OR #6 OR #9
11. ("severe acute respiratory syndrome coronavirus 2" OR "COVID-19" OR "COVID19" OR "COVID2019" OR "2019 ncov" OR "2019-nCoV" OR "2019nCoV" OR "SARS COV 2" OR "SARS-CoV-2" OR "SARS2" OR "hcov19" OR "hcov2019" OR "hcov-19" OR "hcov-2019" OR "wuhan coronavirus*" OR "coronavirus disease 2019" OR "coronavirus disease-19" OR "SARS2" OR "novel coronavirus*" OR "novel corona virus*" OR "new coronavirus*" OR "new corona virus*" **OR "COVID 2019"**).mp
12. #10 AND #11 AND date range 2020 only

**WHO Database**

(("Breastfe*" OR "breast fe*" OR "breastmilk" OR "breast milk" OR "human milk" OR "maternal milk" OR "prelacteal feed*" OR "lactati*" OR "colostrum" OR "mixed feeding" OR "**mother’s milk**" **OR** "**expressed milk**" **OR** "**milk bank***") OR ("pregnan*" OR "gestat*" OR "perinatal" OR "antenatal" OR "parturi*" OR "mother*" OR "infant*" OR "maternal" OR "baby" OR "babies" OR "newborn*" OR "neonat*") OR ("vertical transmission" OR "postnatal transmission" OR "post natal transmission" OR "maternal-infant transmission" OR "adult-to-child" OR "maternal-to-child" OR "mother-to-child" OR "MTCT" OR "PMTCT"))

AND

("severe acute respiratory syndrome coronavirus 2" OR "COVID-19" OR "COVID19" OR "COVID2019" OR "2019 ncov" OR "2019-nCoV" OR "2019nCoV" OR "SARS COV 2" OR "SARS-CoV-2" OR "SARS2" OR "hcov19" OR "hcov2019" OR "hcov-19" OR "hcov-2019" OR "wuhan coronavirus*" OR "coronavirus disease 2019" OR "coronavirus disease-19" OR "SARS2" OR "novel coronavirus*" OR "novel corona virus*" OR "new coronavirus*" OR "new corona virus*" **OR "COVID 2019"**)
